# Plasma Protein Carbonylation in Haemodialysed Patients: Focus on Diabetes and Gender

**DOI:** 10.1155/2018/4149681

**Published:** 2018-07-02

**Authors:** Graziano Colombo, Francesco Reggiani, David Cucchiari, Emanuela Astori, Maria L. Garavaglia, Nicola M. Portinaro, Nicola Saino, Silvia Finazzi, Aldo Milzani, Salvatore Badalamenti, Isabella Dalle-Donne

**Affiliations:** ^1^Department of Biosciences, Università degli Studi di Milano, Via Celoria 26, I-20133 Milan, Italy; ^2^Humanitas Clinical and Research Center-Nephrology Unit, Via Manzoni 56, I-20089 Rozzano Milan, Italy; ^3^Humanitas Clinical and Research Center-Clinica Ortopedica e Traumatologica, Via Manzoni 56, I-20089 Rozzano, Milan, Italy; ^4^Department of Environmental Science and Policy, Università degli Studi di Milano, Via Celoria 26, I-20133 Milan, Italy

## Abstract

Patients with end-stage renal disease (ESRD) undergoing haemodialysis (HD) experience oxidative/carbonyl stress, which is postulated to increase after the HD session. The influence of diabetes mellitus and sex on oxidation of plasma proteins in ESRD has not yet been clarified despite that diabetic nephropathy is the most common cause of ESRD in developed and developing countries and despite the increasingly emerging differences between males and females in epidemiology, pathophysiology, clinical manifestations, and outcomes for several diseases. Therefore, this study aimed to evaluate the possible effect of type 2 diabetes mellitus, gender, and dialysis filter on plasma level of protein carbonyls (PCO) in ESRD patients at the beginning and at the end of a single HD session. Results show that mean post-HD plasma PCO levels are significantly higher than mean pre-HD plasma PCO levels and that the type of dialysis filter and dialysis technique are unrelated to plasma PCO levels. The mean level of plasma PCO after a HD session increases slightly but significantly in nondiabetic ESRD patients compared to diabetic ones, whereas it increases more markedly in women than in men. These novel findings suggest that women with ESRD are more susceptible than men to oxidative/carbonyl stress induced by HD.

## 1. Introduction

Compared to the general population, patients with chronic kidney disease (CKD) are at higher risk for cardiovascular disease (CVD) because of higher prevalence of traditional (such as diabetes mellitus, left ventricular hypertrophy, dyslipidaemia, hypertension, and obesity) and nontraditional cardiovascular risk factors. The latter include uraemia, anaemia, inflammation, and oxidative stress, which all together form part of the malnutrition-inflammation complex (or cachexia) syndrome, which is a strong predictor of morbidity and mortality in these patients. [1–7]. The subgroup of CKD patients that undoubtedly experience the highest degree of oxidative stress is constituted by those patients with end-stage renal disease (ESRD) undergoing haemodialysis (HD). Oxidative stress in ESRD derives from both enhanced oxidative capacity, which is at least partly due to systemic (micro)inflammation and upregulation of superoxide-producing enzymes [5, 8] and diminished antioxidant defences, the latter including impaired enzyme activities and decreased levels of antioxidant vitamins C and E [5, 8–10]. Depletion of circulating antioxidant vitamins in ESRD may originate from diet restriction, reduced absorption, uraemia-related alterations of metabolic pathways, and intradialytic losses [10].

In haemodialysed patients, oxidative stress, which may act synergistically with inflammation, is involved in the development of long-term complications such as amyloidosis, atherosclerosis, and CVD [1, 3, 11]. Plasma biomarkers of protein oxidation in ESRD patients on maintenance HD were measured as indicator of oxidative stress in several studies. Plasma protein oxidation is highlighted by decreased protein thiols [12, 13], which might result from the formation of mixed disulphides between protein thiols and low molecular mass aminothiols (*S*-thiolation). *S*-thiolated plasma proteins, measured as protein thiolation index (PTI) [14], are indeed increased in ESRD patients on maintenance HD [12, 13]. Other biomarkers of protein oxidation in haemodialysed patients are plasma protein-bound dityrosines [15, 16] as well as plasma protein carbonyls (PCO), whose levels are elevated compared to healthy subjects [17–19]. Similarly, biomarkers of inflammation are elevated in haemodialysed patients [20–22].

While it appears that oxidative stress in haemodialysed patients may result from uraemia per se [1, 23], the HD procedure itself may contribute to oxidative/carbonyl stress. Indeed, a few studies showed that plasma PCO levels were significantly higher at the end of a single HD session than before it [17–19, 24, 25]. A prospective cohort study demonstrated that initiation of maintenance HD procedure does not have significant influence on serum levels of the inflammation biomarkers, C-reactive protein, interleukin-6, and interleukin-10, as well as plasma PCO [26]. Otherwise, levels of pentraxin-3, an inflammation biomarker belonging to the same protein family of C-reactive protein (pentraxins), which is rapidly produced locally to the site of inflammation by several cell types and released by neutrophils upon stimulation, were significantly increased at the end of the HD session [22]. This finding suggests that the HD procedure is an exacerbating factor for both oxidative/carbonyl stress and inflammation, presumably due to the activation of neutrophils upon contact with the dialysis filter [11]. Hence, it is postulated that oxidative/carbonyl stress increases in ESRD patients after the HD session.

Only a few studies have focused specifically on the impact of a single HD session on plasma PCO levels in ESRD patients [17–19, 24, 25]. Moreover, the relative importance of diabetes mellitus and sex on plasma PCO levels in haemodialysed patients remains poorly defined and with conflicting results [26–28]. This despite that diabetic nephropathy, alone or in combination with hypertensive nephropathy, is the most common cause of ESRD in developed and developing countries and despite the increasingly emerging differences between male and female in epidemiology, pathophysiology, clinical manifestations, and outcomes for several diseases, among which those displaying oxidative stress-mediated inflammation, such as CVD [29–31]. However, whether sex differences exist with respect to biomarkers of oxidative stress before and after a single HD session in ESRD patients is largely unknown. Therefore, we determined the plasma PCO levels in each individual HD patient before and after a single HD session. This was done by dividing the HD population into groups based on the cooccurrence of type 2 (non-insulin-dependent) diabetes mellitus and the gender. Moreover, the HD population was divided into groups also based on the dialysis filter used, in order to ascertain eventual differences attributable to filter characteristics and, therefore, to the different HD techniques. In fact, there is some evidence indicating that techniques that combine diffusion and convection, such as online haemodiafiltration, may reduce oxidative stress improving the haemodynamic tolerance and the clearance of uremic toxins [32, 33]. However, the effect of convective transport on oxidative stress needs a stronger confirmation.

## 2. Materials and Methods

### 2.1. Study Design and Participants

The study was approved by the institutional review board before initiation and carried out according to the Code of Ethics of the World Medical Association (Declaration of Helsinki). All the 69 Caucasian patients enrolled in the study belong to stage 5 of CKD and are referred to as ESRD patients on maintenance HD. In addition to HD, patients are treated with a pharmacological treatment that varies upon the clinical necessities and consists mainly on the treatment of ESRD complications. Most of the patients assume drugs for anaemia (i.e., iron intravenous supplementation and/or erythropoietin) and bone mineral disorder (i.e., calcium supplementation, phosphate binders, vitamin D, paricalcitol, and/or calcimimetics). In addition, patients may also take specific drugs for other comorbidities, for example, hypertension, diabetes mellitus, ischemic cardiopathy, and other vasculopathies. Blood samples were collected, after informed written consent, from ESRD patients undergoing maintenance HD at the Nephrology and Dialysis Unit of the Humanitas Clinical and Research Center (Rozzano, Milan, Italy). The samples were collected at the arterial line at the beginning and at the end of the HD session. The presence of a clinically overt infectious process was the only exclusion criteria. For every patient, an anamnestic record was collected. A deidentification of the samples was performed before any additional data processing. The baseline clinical characteristics of recruited patients are shown in Table 1. Control blood samples were collected from 20 (10 males and 10 females) age-matched voluntary healthy donors at the Analysis Laboratory of the University of Milan (Laboratorio Analisi, Università degli Studi di Milano) after obtaining informed verbal consent. Criteria included no known history of CKD or other diseases that could influence the analysis. In particular, healthy subjects were tested for serum creatinine in order to exclude CKD.

### 2.2. Dialysis Filters

We used different dialysis filters, all characterised by high-flux (defined as a *β*2-microglobulin clearance of over 20 ml/min) synthetic biocompatible membranes. In particular, the filters used are the following, classified by the dialysis technique:

1. Diffusive technique (standard bicarbonate haemodialysis) (*n* = 41)
  - Revaclear™ 300, whose membrane is made of polyarylethersulphone (PAES) + polyvinylpyrrolidone (PVP) (surface: 1.4 m^2^, thickness: 35 *μ*m), for 23 patients
  - Revaclear™ 400, whose membrane is made of PAES + PVP (surface: 1.8 m^2^, thickness: 35 *μ*m), for 14 patients
  - Filtryzer® 1.6, whose membrane is made of polymethyl metacrylate (PMMA) (surface: 1.6 m^2^, thickness: 30 *μ*m), for 4 patients
2. Diffusive plus convective techniques (online haemodiafiltration (online HDF) and acetate-free biofiltration (AFB)) (*n* = 27)
  - Polyflux™ 170 H, whose membrane is made of PAES + PVP + polyamide (PA) (surface: 1.7 m^2^, thickness: 50 *μ*m), for 14 patients
  - Polyflux™ 210 H, whose membrane is made of PAES + PVP + PA (surface: 2.1 m^2^, thickness: 50 *μ*m), for 11 patients
  - Nephral™ ST 400, whose membrane is made of acrylonitrile/methallyl sulphonate copolymer, coated with high-molecular-weight polyethyleneimine (PEI) (surface: 1.65 m^2^, thickness: 42 *μ*m), for one patient
  - Nephral™ ST 500, whose membrane is made of acrylonitrile/methallyl sulphonate copolymer, coated with PEI (surface: 2.15 m^2^, thickness: 42 *μ*m), for one patient

Filters a, b, and d–g are produced by Gambro®-Baxter, whereas filter c is produced by Toray Industries Inc.

### 2.3. Sample Collection

Venous blood samples of 10 ml were collected from ESRD patients before HD session, and 5 ml were obtained after the same session. All samples were collected on the long interdialytic interval, that is, two days apart from the previous HD session. Blood was taken from the arteriovenous fistula or central venous catheter. K_3_EDTA was used as anticoagulant in all the blood samples. From healthy donors, 10 ml of venous blood was collected from the antecubital vein. All the samples were processed within the first hour from blood sampling through centrifugation for 10 min at 1000*g*, obtaining pre-HD and post-HD plasma aliquots from ESRD patients and plasma aliquots from healthy subjects. Such aliquots were stored at −80°C until the execution of the assays.

### 2.4. Detection of Plasma Protein Carbonylation by SDS-PAGE and Western Blot

Plasma proteins were fractionated on 12.5% (*w*/*v*) reducing SDS-PAGE gels and electroblotted onto a polyvinylidene difluoride (PVDF) membrane. Protein carbonylation was detected, after derivatization with DNPH, with anti-DNP antibodies specific for the 2,4-dinitrophenyl hydrazone-carbonyl adduct by Western blot immunoassay as previously reported [34, 35]. Immunoreactive protein bands were visualized by enhanced chemiluminescence (ECL). Protein bands on PVDF membranes were then visualized by washing the blots extensively in PBS and then staining with Ponceau Red.

### 2.5. Determination of Plasma Protein Carbonyls by Enzyme-Linked Immunosorbent Assay (ELISA)

Plasma PCO were measured using the ELISA kit manufactured by Enzo Life Sciences (ALX-850-312-KI01). Carbonylated protein standard (40 mg/ml containing 0-0.12-0.22-0.42-0.7-0.9 nmol carbonyls/mg protein) and human plasma samples (60–75 mg/ml) were diluted 1 : 40 in DNPH solution and incubated 45 min to allow PCO derivatization. A 1 : 200 dilution in ELISA buffer was then performed before adding 200 *μ*L (1-2 *μ*g of protein) in each ELISA plate well. We incubated ELISA plate overnight at 4°C to allow protein binding. ELISA assay was performed according to the manufacturer's instructions. Absorbance of plate wells was read at 450 nm using the Plate Reader TECAN Infinite® 200 PRO. In all the performed assays, calibration line showed an R^2^ close to 0.99. We then calculated carbonyl content of samples by using the regression factors (intercept with the *y*-axis and line slope) obtained from standard curve.

### 2.6. Determination of Clinical Laboratory Parameters

Creatinine, C-reactive protein, white blood cell count, albumin, fibrinogen, haemoglobin, ferritin, total iron-binding capacity, urea, sodium, potassium, calcium, and phosphorus were measured *by* standardized methods at the clinical laboratory of the Humanitas Clinical and Research Center [12, 16].

### 2.7. Statistical Analysis

The paired Student's *t*-test was used to test whether differences in plasma PCO level in ESRD patients before (pre-HD) and after (post-HD) a single HD session were significant. The paired Student's *t*-test was also used to test for differences in plasma PCO levels before and after a single HD session by dividing the haemodialysed patients depending on the dialysis filter used, the cooccurrence of type 2 diabetes mellitus, and the gender. All the values are expressed as mean and standard errors (SE). A *p* value <0.05 was considered to be significant. The statistical significances are marked as ^∗^ = *p* < 0.05 and ^∗∗^ = *p* < 0.01. The relationship between pre-HD and post-HD PCO levels was investigated by simple linear regression analysis.

## 3. Results

**Figure 1:**
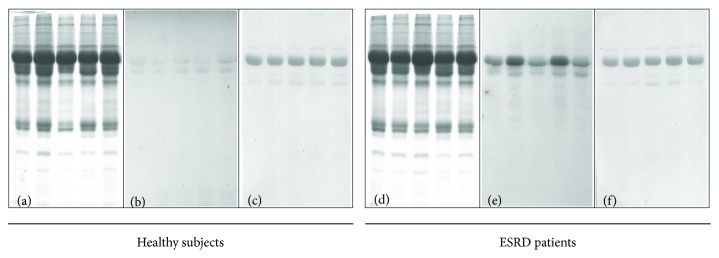
Plasma protein carbonylation. Representative SDS-PAGE (a and d) and Western blot with anti-DNP antibody developed with ECL (b and e) of plasma proteins in age-matched voluntary healthy subjects (a and b) and in five representative ESRD patients on maintenance HD (d and e). Visualization of proteins in PVDF membrane with Ponceau Red staining (c and f).

PCO are considered the most general and the most commonly used biomarkers of severe oxidative protein damage. The results of protein carbonylation assessed by Western blotting using anti-DNP antibodies from five ESRD patients and five age-matched voluntary healthy donors are presented in Figure 1. Plasma proteins from healthy subjects showed a very low level of carbonyl content (Figure 1(b)), whereas plasma proteins from ESRD patients clearly exhibited an increase in carbonyl content (Figure 1(e)). We applied reversible Ponceau Red staining to assess equal loading of gels (Figures 1(c) and 1(f)).

**Figure 2:**
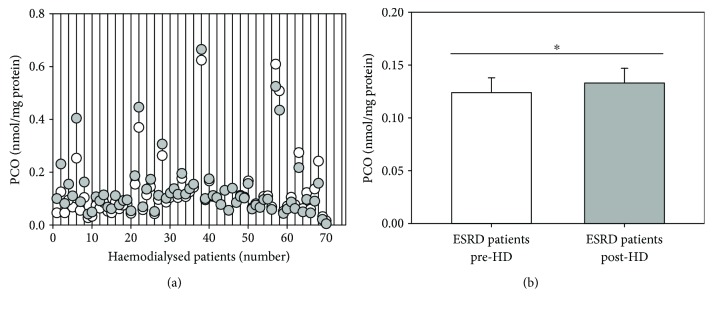
Effect of a single HD session on the level of plasma PCO in ESRD patients on maintenance HD. (a) Scatter diagram showing plasma PCO level in individual haemodialyzed patients immediately before (white circles) and after (grey circles) a single HD session. (b) Histograms showing the mean plasma PCO level in MHD patients immediately before (pre-HD) and after (post-HD) a single HD session. Data are expressed as the mean ± SE. ^∗^*p* < 0.05.

We also determined the effect of HD on the plasma PCO levels, measured by a sensitive ELISA method [36, 37], in each individual ESRD patient at the beginning and at the end of the HD session. In this regard, it is important to note that we had previously shown that the total plasma protein concentration in ESRD patients increases significantly after the HD session due to net volume ultrafiltration [12]. Therefore, in this study, plasma PCO are expressed as nmol/mg protein. Scatter diagram of plasma PCO levels in haemodialyzed patients is shown in Figure 2(a). In most ESRD patients, we observed a small increase in the plasma PCO level after the HD procedure compared to the pre-HD value. Differently, some ESRD patients showed the same or a slightly lower plasma PCO level immediately after the HD session compared to the pre-HD value. The result of the paired Student's *t*-test applied to the mean value of plasma PCO level measured in ESRD patients pre-HD (mean 0.1239 ± 0.0140 nmol/mg protein) and post-HD (mean 0.1332 ± 0.0140 nmol/mg protein) proved that the means are significantly different (*p* < 0.05) (Figure 2(b)). There were no differences between patients showing an increase as compared to those showing no change or a slight decrease in plasma carbonyl levels after dialysis. In particular, we did not notice any difference in chronological age, HD vintage, body mass index, WBC count and concentration of C-reactive protein, albumin, fibrinogen, haemoglobin, urea, creatinine, sodium, potassium, calcium, phosphorus, and ferritin (not shown).

**Figure 3:**
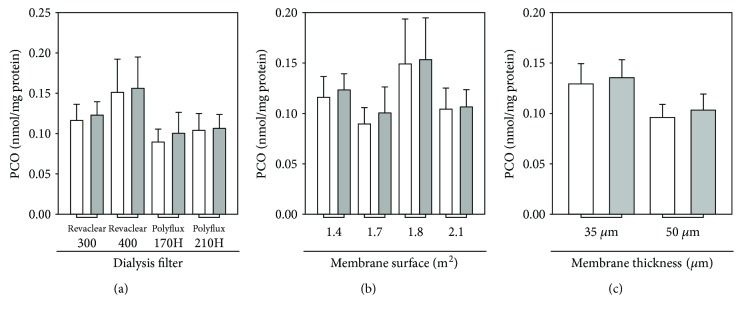
Influence of the dialysis filter type on the level of plasma PCO in ESRD patients on maintenance HD. Haemodialyzed patients (*n* = 62) were divided into subgroups based on the characteristics of the dialysis filters used during the HD session: (a) filter type (i.e., Revaclear™ 300, Revaclear 400, Polyflux™ 170 H, and Polyflux 210 H), (b) membrane surface area, and (c) membrane thickness. In all panels, histograms show the plasma PCO level in haemodialysed patients immediately before (pre-HD, white bars) and after (post-HD, grey bars) a single HD session. For filter details, see the Materials and Methods section. The seven remaining ESRD patients, who were dialyzed with Filtryzer 1.6, Nephral™ ST 400, or Nephral ST 500 filters, were not included because their number is too low to make a reliable statistical analysis. Data are expressed as the mean ± SE.

**Figure 4:**
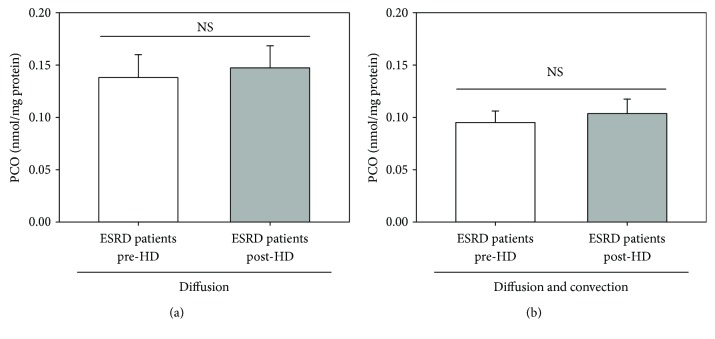
Influence of the dialysis technique (diffusive versus diffusive plus convective) on the level of plasma PCO in ESRD patients on maintenance HD. Haemodialyzed patients were divided into subgroups based on the dialysis technique used during the HD session: (a) diffusive technique (standard bicarbonate haemodialysis) (*n* = 41), (b) diffusive plus convective techniques (online HDF) and acetate-free biofiltration (*n* = 28). In both panels, histograms show the plasma PCO level in haemodialysed patients immediately before (pre-HD, white bars) and after (post-HD, grey bars) a single HD session. Data are expressed as the mean ± SE. In panel (a), *p* = 0.0880; in panel (b), *p* = 0.2188.

In HD, patient's blood is allowed to flow through a filter (the haemodialyser), whereby waste products and excess water are removed across a semipermeable membrane separating flowing blood from the dialysate stream. The clean blood is then returned to the haemodialysed patient's body, while wastes are discharged. The main determinant of the quality of HD therapy is represented by the artificial membrane packed into the haemodialyser. The HD therapy per se, in particular the type of dialysis membrane, contributes to the increased production of ROS in ESRD patients [38]. Indeed, typically, an HD patient's blood is in contact with the synthetic HD membrane for a ∼3.5 to 4 h/session and three sessions/a week. This prolonged contact of blood with the synthetic polymer surface results in two long-term complications, namely membrane-induced oxidative stress and membrane-induced inflammation, both of which contribute to CVD development [39]. Therefore, the dialysis filter may have a potential relevant impact on plasma PCO levels post-HD. Sixty-two out of 69 ESRD patients recruited in the study were dialyzed with filters made (mainly) of PAES + PVP that differ in extension and thickness (see Materials and Methods). Therefore, we measured the plasma PCO level immediately before and after the HD session by subdividing those 62 ESRD patients based on the characteristics of the filter membrane or the dialysis technique (diffusive versus diffusive plus convective) used during the HD session. As shown in Figures 3 and 4, the comparison of filter type (Figure 3(a)), membrane surface area (Figure 3(b)), and membrane thickness (Figure 3(c)) as well as dialysis technique (Figure 4) did not reveal any statistically significant difference in plasma PCO levels before (pre-HD) and after (post-HD) the HD session.

**Figure 5:**
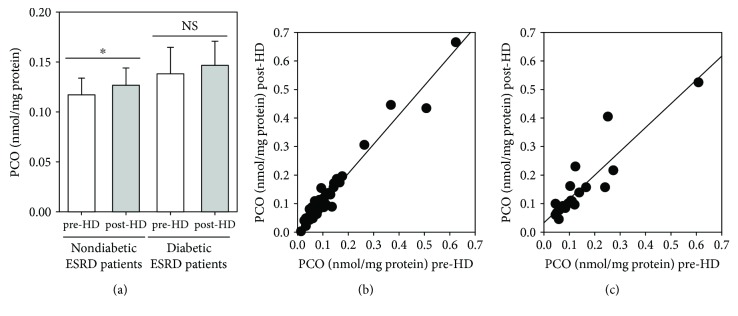
Plasma PCO levels measured in nondiabetic and diabetic ESRD patients on maintenance HD. (a) Histogram showing the plasma PCO level in nondiabetic and diabetic haemodialyzed patients measured immediately before (pre-HD, white bars) and after (post-HD, grey bars) a single HD session. Data are expressed as the mean ± SE. (b) Regression analysis showing the relation between pre-HD and post-HD plasma PCO levels in nondiabetic haemodialyzed patients. Slope of the line is *a* = 1.0154. (c) Regression analysis showing the relation between pre-HD and post-HD plasma PCO levels in diabetic haemodialyzed patients. Slope of the line is *a* = 0.8349. ^∗^*p* < 0.05.

Considering that diabetes mellitus occurs as an important comorbidity in the ESRD population (often composed predominantly of subjects in advanced age) and that some studies suggest that oxidative stress in diabetic patients leads to increased plasma PCO levels [40, 41], we hypothesized that diabetic ESRD patients could experience a significant increased oxidative stress in comparison with nondiabetic ESRD patients. Therefore, we evaluated the plasma PCO levels immediately before and after a single HD session by subdividing all ESRD patients into diabetics (*n* = 22; mean age: 71.6 ± 2.1 yrs; HD vintage: 5.1 ± 0.7 yrs) and nondiabetics (*n* = 47; mean age: 67.8 ± 2.0 yrs; HD vintage: 6.1 ± 0.6 years) (Figure 5). Data were analysed according to a paired sample *t*-test used to compare means of pre- and post-HD plasma PCO level in each of the two groups of haemodialyzed patients. The results proved that the means pre-HD and post-HD are significantly different in nondiabetics (0.1172 ± 0.0166 nmol/mg protein and 0.1268 ± 0.0172 nmol/mg protein, resp., *p* < 0.05), whereas they are not significantly different in diabetics (0.1382 ± 0.0264 nmol/mg protein and 0.1467 ± 0.0244 nmol/mg protein, resp.) (Figure 5(a)); in addition, differences in both pre-HD and post-HD plasma PCO levels between diabetics and nondiabetics are not statistically significant (*t*-test for independent samples). Pre-HD plasma PCO levels were significantly positively correlated with post-HD plasma PCO concentrations both in nondiabetic (*r* = 0.9766, *p* < 0.0001) (Figure 5(b)) and diabetic (*r* = 0.9033, *p* < 0.0001) (Figure 5) ESRD patients.

**Figure 6:**
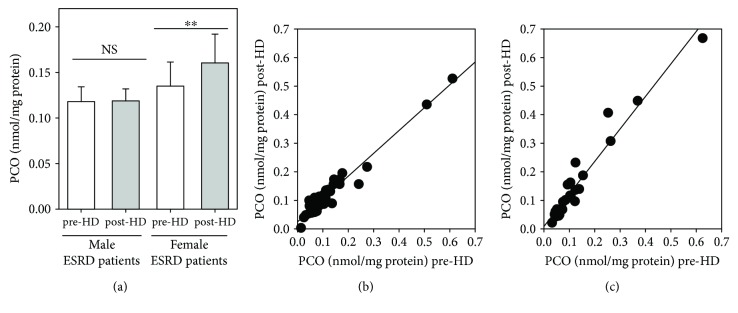
Plasma PCO levels evaluated in male and female ESRD patients on maintenance HD. (a) Histogram showing the plasma PCO levels in male and female ESRD patients measured immediately before (pre-HD, white bars) and after (post-HD, grey bars) a single HD session. Data are expressed as the mean ± SE. (b) Regression analysis showing the relation between pre-HD plasma PCO levels and post-HD plasma PCO levels in male ESRD patients. Slope of the line is *a* = 0.7993 (c) Regression analysis showing the relation between pre-HD plasma PCO levels and post-HD plasma PCO levels in female ESRD patients. Slope of the line is *a* = 1.1383. ^∗∗^*p* < 0.01.

We also evaluated the plasma PCO levels immediately before and after a single HD session by separating ESRD patients on HD by gender (males: *n* = 45, mean age 70.1 ± 2.4 yrs, HD vintage 5.6 ± 0.6 yrs; females: *n* = 24, mean age 66.9 ± 3.1 yrs; HD vintage 6.1 ± 0.8 yrs) (Figure 6). Data were analysed according to a paired sample *t*-test used to compare means of pre- and post-HD plasma PCO level in each of the two groups of haemodialysed patients. The results proved that the means pre-HD and post-HD are not significantly different in males (0.1180 ± 0.0163 nmol/mg protein and 0.1187 ± 0.0134 nmol/mg protein, resp.), whereas they are significantly different in females (0.1348 ± 0.0267 nmol/mg protein and 0.1604 ± 0.0313 nmol/mg protein, resp., *p* < 0.01) (Figure 6(a)); in addition, differences in both pre-HD and post-HD plasma PCO levels between men and women are not statistically significant (unpaired *t*-test). Pre-HD plasma PCO levels were significantly positively correlated with post-HD plasma PCO concentrations both in male (*r* = 0.9730, *p* < 0.0001) (Figure 6(b)) and female (*r* = 0.9702, *p* < 0.0001) (Figure 5(c)) ESRD patients.

## 4. Discussion

Protein carbonylation, which may result from direct oxidation of lysine, arginine, proline, and threonine residues and interaction with reactive carbonyl species produced from carbohydrate and lipid oxidation or non-oxidative reactions with dicarbonyl compounds, is an indicator of oxidative protein damage [42]. Its use as a biomarker of oxidative stress has some advantages because of the stability of PCO in comparison with other oxidation products. We showed that plasma proteins of ESRD patients on HD exhibited an increase in carbonyl content compared to plasma proteins of the controls, which was especially evident in albumin (Figure 1). These results are in agreement with a previous study, which showed that post-HD plasma PCO levels are significantly increased compared to pre-HD levels and that carbonylation affects almost a dozen of plasma proteins, among which albumin is the most susceptible to carbonyl formation [24]. In this regard, it is interesting to note that following carbonylation, albumin vasculoprotective effects in haemodialysed patients are impaired [43] and, therefore, carbonylated albumin may play a role in the early atherogenic events of chronic uraemia by directly damaging the endothelium [24].

The increased levels of PCO measured in haemodialysed patients suggest that protein carbonylation may be an important biomarker of oxidative stress in ESRD patients as well. A number of studies compared biomarkers of oxidative stress between ESRD patients and age-matched healthy subjects [19, 28, 44] or between CKD patients with different CKD stages [45–47]. Here, we compared plasma levels of PCO of each patient before and after a single HD session. We found that mean post-HD levels of plasma PCO are significantly higher than mean plasma PCO levels before the HD session (Figure 2). These results are in agreement with previous ones showing that the levels of plasma PCO were significantly higher at the end of a single HD session than before it [17–19, 24, 25, 48], whereas they differ from those of a unique study that did not report any increase in plasma PCO level after HD [49] (Table 2).

About the possible influence of the dialysis membrane on PCO levels, we have considered that during the HD session, the contact of blood with the dialysis membrane and the loss of antioxidants may promote oxidative stress. In a study conducted on 15 nondiabetic ESRD patients (9 males and 6 females), PCO levels were found to be significantly increased after a HD session with a cuprophane membrane [50] and increased, but not significantly, after a HD session with a polysulfone membrane [50]. This data is intuitive, as hydrophilic cuprophane membranes are known to severely activate complement and leukocytes [51]. In addition, proteomic investigations suggest that dialysis membranes may retain, at least in part, plasma proteins, especially carbonylated ones [52, 53]. This would occur mainly via the protein adsorptive properties of the membrane material [54], since the main mechanisms for solute removal during HD, diffusion, and convection have poor ability to remove high molecular weight solutes, such as proteins. Most of haemodialysed patients recruited into the study were dialyzed using membranes made of PAES and PVP ± PA that differ in surface and thickness (see Materials and Methods). In this regard, neither dialysis membrane composition, surface, and thickness (Figure 3) nor dialysis technique (Figure 4) significantly affected pre-HD and post-HD levels of plasma PCO. Therefore, the membrane surface area and thickness of the dialysis filter as well as the dialysis technique do not significantly affect the observed increase in PCO during the HD session. It is interesting to note that some evidence suggests a progressive significant decrease in concentration of inflammatory biomarkers [55] and advanced oxidation protein products [56] when vitamin E-coated polysulfone membranes are used for the dialysis sessions. This might suggest a protective effect of vitamin E-coated polysulfone membrane against inflammation and oxidative stress in haemodialysed patients.

It is well known that oxidative stress is increased in diabetes mellitus. In particular, some studies have shown that plasma PCO levels are increased in diabetics [40, 41]. Unfortunately, the combination of CKD and diabetes is associated with increased morbidity and mortality, mainly due to increased cardiovascular risk [57]. About one-third (32%) of the haemodialysed patients recruited in our study are diabetics. Hence, we compared plasma PCO levels before and after a single HD session in diabetic and nondiabetic ESRD patients (Figure 5). Unexpectedly, pre-HD PCO levels are not significantly different in nondiabetic and diabetic ESRD patients nor are the post-HD ones. Nevertheless, the mean value of plasma PCO levels increases slightly but significantly in nondiabetic ESRD patients after the HD session. Differently, the mean values of pre-HD and post-HD plasma PCO levels are not significantly different in diabetic ESRD patients. These findings could suggest that the HD session induces a moderate increase in oxidative/carbonyl stress. Therefore, nondiabetic ESRD patients seem to be more susceptible to oxidative stress induced by the HD session. These results are only partially consistent with those described by Dursun and colleagues [58], who determined the levels of several biomarkers of oxidative stress in 20 nondiabetic ESRD patients (9 males and 11 females) and 20 diabetic ESRD patients (9 males and 11 females) before and after HD. They concluded that both diabetes and HD increase oxidative stress and that their combined effect on oxidative stress is greatest in diabetic ESRD patients. However, a limitation of their study was the very small number of subjects.

In contrast to what is observed in the general population, where females have a longer life expectancy than males [59], female ESRD patients have as poor survival as male ESRD patients [60, 61]. These observations are somewhat surprising considering that haemodialysed women have a lower prevalence of CVD [62] are less likely to develop left-ventricular hypertrophy [63] and are less predisposed to cardiovascular calcification [64]. It has been suggested that noncardiovascular mortality is the main explanation for the loss of the survival advantage in ESRD women on HD [62]. Moreover, the HD procedure too may contribute to cancel out the survival advantage in ESRD women [60]. So, we compared plasma PCO levels before and after a single HD session in male and female ESRD patients (Figure 6). In men, the mean values of pre-HD and post-HD plasma PCO levels are not significantly different, whereas in women, the mean value of post-HD plasma PCO level is significantly higher than that of pre-HD. These novel findings suggest that haemodialysed women seem to be more susceptible to oxidative stress induced by the HD session.

In summary, all these results suggest that (i) the HD session increases plasma protein carbonylation; therefore, although important advances have been done in the field of dialysis biocompatibility, the HD session probably still represents a source of oxidative stress; (ii) plasma PCO level measurement may become an indicator of oxidative/carbonyl stress in ESRD and could be included in the routine monitoring of haemodialysed patients, since standard uraemia and inflammation biomarkers may not be sufficient on their own to describe the inflammatory/oxidative state of ESRD patients on HD; and (iii) haemodialysed women seem to be more susceptible to oxidative/carbonyl stress induced by the HD session than men. On the basis of these findings, it seems appropriate to suggest that the female sex could be considered a fundamental biologic variable (or a “risk factor”) associated with HD-induced plasma protein carbonylation in ESRD patients on maintenance HD. Therefore, this study shows that gender differences exist in plasma PCO levels of haemodialysed patients before and after a single HD session and highlights the critical importance of reporting of sex information in study description, data analyses, results, and their interpretation in basic science and medical/clinical research studies concerning ESRD. As a matter of fact, the prevalence of CKD stages 1–5 among US adults aged 18 years or older is higher in women than men (16% versus 13%); however, men are 64% more likely than women to develop ESRD [65], yet most studies of ESRD group together men and women and assume any underlying pathophysiology is the same. So, this study can contribute, in its own small way, to increase our understanding of the gender differences of diseases, in particular in the field of nephrology, where some gender differences have been documented [66]. Indeed, women seem to be somewhat protected from developing ESRD [67]. In addition, the cumulative incidence of ESRD is low during the reproductive ages and begins to rise ten years later in women than in men among participants in community-based screenings. Moreover, the mean age at the start of HD is also higher in women than in men [67].

Finally, our study has some limitations. Firstly, it includes a relatively small number of ESRD male and female patients and has been performed in only one single HD center. Secondly, the majority of the patients studied in this cohort are from Italy and of Caucasian race, and the applicability of the study findings across nationalities and races remains unclear. However, we hope that these results stimulate further research with a larger number of men and women on HD recruited from different dialysis centers, possibly of different nationalities and races, to advance our understanding of the pathophysiology of sex (and possibly of nationality and/or race) differences in CKD and improve clinical care of women with CKD. While some advances have been made in both clinical and basic research, much remains poorly understood, both at the molecular and clinical levels.

## 5. Conclusions

Post-HD plasma PCO level increases in nondiabetic but not in diabetic ESRD patients, more markedly in women than in men. Women with ESRD are more susceptible than men to HD-induced oxidative/carbonyl stress.

## Figures and Tables

**Table 1:** Characteristics of haemodialysed patients with ESRD. Data are expressed as mean ± standard deviation.

	Haemodialysed patients (*n* = 69)	Reference range
Age (years)	69.0 ± 1.5	—
Dialysis vintage (years)	5.8 ± 0.46	—
Sex	45 male, 24 female	—
Diabetes mellitus	47 nondiabetic, 22 diabetic	—
Creatinine (mg/dl)	9.24 ± 0.35	0.6–1.3
Urea (mg/dl)	148.98 ± 4.55	10.00–50.00
C-reactive protein (mg/dl)	0.55 ± 0.08	0.01–1
Albumin (g/dl)	3.5 ± 0.04	3.5–5
White blood cells (cells/mm^3^)	7257.97 ± 271.06	4 · 10^3^
Haemoglobin (g/dl)	11.03 ± 0.12	13–18
Sodium (mmol/l)	137.80 ± 0.38	135–145
Potassium (mmol/l)	5.26 ± 0.09	3.5–5.1
Calcium (mmol/l)	2.22 ± 0.02	2.1-2.6
Phosphorus (mmol/l)	1.63 ± 0.05	0.8–1.5
Ferritin (ng/ml)	201.26 ± 16.85	20–250

**Table 2:** Studies that examined the plasma PCO levels in haemodialysed patients before haemodialysis (pre-HD) and after haemodialysis (post-HD).

Study	HD group number (age and sex) and dialysis vintage	Control group number (age and sex)	PCO HD group	PCO control group
Ward et al. [17]	22 HD patients (age 51 ± 5 years, 8 M and 4 F). Divided into two groups: 11 patients treated with polysulfone membrane 11 patients treated with cellulose triacetate membrane Dialysis vintage 49 ± 11 months	17 healthy subjects (age range 23–54 years, both M and F)	Polysulfone membrane pre-HD 0.144 ± 0.037 mmol/mg protein post-HD 0.175 ± 0.029 mmol/mg protein *p* < 0.05 Cellulose triacetate membrane pre-HD 0.145 ± 0.030 mmol/mg protein post-HD 0.178 ± 0.035 mmol/mg protein *p* < 0.05	0.041 ± 0.008 mmol/mg protein
Dursun et al. [48]	20 HD patients (age and sex unspecified) Dialysis vintage unspecified	20 healthy subjects (age and sex unspecified)	Pre-HD 0.889 ± 0.063 nmol/mg protein post-HD 0.997 ± 0.066 nmol/mg protein *p* < 0.05	0.417 ± 0.036 nmol/mg protein
Pieniazek et al. [18]	10 HD patients (mean age 58 ± 11 years, sex unspecified) Dialysis vintage unspecified	9 healthy subjects (age 46 ± 15 years, sex unspecified)	Pre-HD 2.27 ± 0.2 mmol/l post-HD 2.94 ± 0.12 mmol/l *p* < 0.0002	0.67 ± 0.07 mmol/l
Terawaki et al. [49]	83 anuric HD patients divided into two groups: patients with CVD (*n* = 66, age 63.5 ± 12.5 years, 32 M and 34 F) Dialysis vintage 85.0 ± 64.6 months patients without CVD (*n* = 20, age 74.3 ± 12.8 years, 11 M and 9 F) Dialysis vintage 58.3 ± 33.3 months	—	patients with CVD pre-HD 0.81 ± 0.16 nmol/mg protein post-HD 0.53 ± 0.13 nmol/mg protein patients without CVD pre-HD 0.82 ± 0.17 nmol/mg protein post-HD 0.58 ± 0.16 nmol/mg protein	—
Albarello et al. [25]	23 HD patients (9 men and 14 women, mean age 50.8 ± 17.3 years) Dialysis vintage unspecified	—	Pre-HD 0.62 ± 0.14 nmol/mg protein post-HD 0.86 ± 0.16 nmol/mg protein *p* < 0.001	—
Caimi et al. [19]	31 HD patients (61.5 ± 12.8 years, 16 men and 15 women) Dialysis vintage 48.5 ± 35.7 months	26 healthy subjects (age 43.54 ± 6.92 years, 17 M and 9 F)	Pre-HD 0.62 ± 0.14 nmol/mg protein post-HD 0.86 ± 0.16 nmol/mg protein *p* < 0.01	0.440 ± 0.134 nmol/mg protein

## Data Availability

The authors are available to share their data.

## Abbreviations

CKD: Chronic kidney disease
CVD: Cardiovascular disease
DNPH: 2,4-Dinitrophenylhydrazine
ELISA: Enzyme-linked immunosorbent assay
ESRD: End stage renal disease
HD: Haemodialysis
PCO: Protein carbonyls
ROS: Reactive oxygen species.
